# The importance of the generation interval in investigating dynamics and control of new SARS-CoV-2 variants

**DOI:** 10.1098/rsif.2022.0173

**Published:** 2022-06-15

**Authors:** Sang Woo Park, Benjamin M. Bolker, Sebastian Funk, C. Jessica E. Metcalf, Joshua S. Weitz, Bryan T. Grenfell, Jonathan Dushoff

**Affiliations:** ^1^ Department of Ecology and Evolutionary Biology, Princeton University, Princeton, NJ, USA; ^2^ Princeton School of Public and International Affairs, Princeton University, Princeton, NJ, USA; ^3^ Department of Biology, McMaster University, Hamilton, Ontario, Canada; ^4^ Department of Mathematics and Statistics, McMaster University, Hamilton, Ontario, Canada; ^5^ M. G. DeGroote Institute for Infectious Disease Research, McMaster University, Hamilton, Ontario, Canada; ^6^ Department for Infectious Disease Epidemiology, London School of Hygiene & Tropical Medicine, London, UK; ^7^ Centre for Mathematical Modelling of Infectious Diseases, London School of Hygiene & Tropical Medicine, London, UK; ^8^ School of Biological Sciences, Georgia Institute of Technology, Atlanta, GA, USA; ^9^ School of Physics, Georgia Institute of Technology, Atlanta, GA, USA; ^10^ Institut de Biologie, École Normale Supérieure, Paris, France

**Keywords:** SARS-CoV-2, COVID-19, generation interval, reproduction number, variants of concern

## Abstract

Inferring the relative strength (i.e. the ratio of reproduction numbers) and relative speed (i.e. the difference between growth rates) of new SARS-CoV-2 variants is critical to predicting and controlling the course of the current pandemic. Analyses of new variants have primarily focused on characterizing changes in the proportion of new variants, implicitly or explicitly assuming that the relative speed remains fixed over the course of an invasion. We use a generation-interval-based framework to challenge this assumption and illustrate how relative strength and speed change over time under two idealized interventions: a constant-strength intervention like idealized vaccination or social distancing, which reduces transmission rates by a constant proportion, and a constant-speed intervention like idealized contact tracing, which isolates infected individuals at a constant rate. In general, constant-strength interventions change the relative speed of a new variant, while constant-speed interventions change its relative strength. Differences in the generation-interval distributions between variants can exaggerate these changes and modify the effectiveness of interventions. Finally, neglecting differences in generation-interval distributions can bias estimates of relative strength.

## 1. Introduction

Estimating variant epidemic strength and speed remains a key question in understanding the threat of SARS-CoV-2 variants of concern (VoCs) [1–8]. Epidemic ‘strength’ is measured by the reproduction number R—a unitless quantity representing the average number of new infections caused by a typical infection. A pathogen can spread in a population if R>1 [9]. The epidemic strength also determines the final size of an epidemic in a homogeneously mixing population under the mass-action assumption [10]. Epidemic ‘speed’ is characterized by the growth rate *r*, which has units of 1/time and describes the exponential rate of pathogen spread at the population level. Like epidemic strength, epidemic speed also determines conditions for pathogen elimination: *r* = 0 is a threshold equivalent to R=1 under constant conditions. However, epidemiological modellers have often over-emphasized R at the expense of *r* [11]; we thus use the terms ‘strength’ and ‘speed’ here to underline our contention that these metrics are better seen as complementary perspectives (and to link them to complementary perspectives on measuring the transmission advantage of new variants).

Epidemic speed is typically estimated from time series of incidence of infection during the exponential growth period [12–14], but epidemic strength is difficult to measure from incidence time series. Instead, epidemic strength is often inferred from the observed epidemic speed using the generation-interval distribution *g*(*τ*), an approach popularized by Wallinga & Lipsitch [15]. The generation interval, defined as the time between infection and transmission, provides information about the time scale of individual-level transmission [16]. The generation interval is also distinctly different from other ‘transmission intervals’ that measure time between successive infections—including the serial interval, which is defined as the time between symptom onsets in an infector–infectee pair [17–21].

The exact shape of the distribution depends on several factors—including the shape of latent and infectious period distributions [22–24] as well as more detailed life history of a pathogen [25]—and thus can be difficult to estimate. While it is possible to consider general forms of generation-interval distributions [26,27], summarizing the distribution in terms of its mean and variability—for example, by assuming they are Gamma distributed—can still provide a robust link between epidemic speed and strength for real pathogens and yield important biological insights [28]. In particular, several studies have noted, in various contexts, that mechanisms that increase the mean generation interval increase the epidemic strength R that would be estimated for a given epidemic speed *r* [29–32].

Analyses of new variants have characterized *relative* strength (i.e. the ratio of reproduction numbers of the invading and resident strains, here called *ρ*) and speed (i.e. the difference between growth rates of the invading and resident strains, here called *δ*). As an example, we consider a new variant invading a wild-type strain in this paper and use $R_{\mathrm{var}}$ and $R_{\mathrm{wt}}$ to denote their respective reproduction numbers and $r_{\mathrm{var}}$ and $r_{\mathrm{wt}}$ to denote their respective growth rates at a given time (and therefore $\rho =R_{\mathrm{var}}/R_{\mathrm{wt}}$ and $\delta =r_{\mathrm{var}}-r_{\mathrm{wt}}$). Many analyses have focused on changes in the *proportion* of a new variant to estimate its relative speed [1–7]. Focusing on proportions can be advantageous, because changes in proportions are less sensitive to changes in testing and to other transient phenomena that would affect variants and wild-type viruses similarly; however, estimates of relative speed from changes in the proportion of a new variant have typically relied on the assumption that the relative speed remains fixed over the time scale of an invasion. Instead (or additionally), some studies have assumed a fixed value of the relative strength and tried to predict relative speed [2,3].

While both approaches are reasonable, holding different quantities constant (i.e. strength or speed) can lead to different conclusions about the spread of the pathogen and its control [11]. To illustrate the differences in conclusions when holding R or *r* fixed, we consider two idealized interventions of constant strength and constant speed. Before these interventions are introduced, the dynamics of pathogen spread can be characterized in terms of the pre-intervention kernel *K*_pre_(*τ*), which represents the rate at which an infected individual generates secondary infections *τ* time units after infection. The pre-intervention kernel can be further decomposed in terms of the epidemic strength R and the generation-interval distribution *g*(*τ*): $K_{\mathrm{pre}}(\tau )=Rg(\tau )$; therefore, the infection kernel integrates to R while the generation-interval distribution integrates to 1. Then, a constant-strength intervention reduces transmission by a constant factor *θ* throughout infection such that the post-intervention kernel is *K*_post_(*τ*) = *K*_pre_(*τ*)/*θ*. In this case, the intervention strength *θ* must be greater than the epidemic strength R to control the epidemic. By contrast, a constant-speed intervention reduces transmission after infection by a constant rate *ϕ* throughout infection: *K*_post_(*τ*) = *K*_pre_(*τ*)exp(−*ϕτ*). In this case, the intervention speed *ϕ* must be greater than the pre-intervention epidemic speed *r* to control the epidemic. We note that the resulting post-intervention generation-interval distribution under a constant-speed intervention is *not* equal to *g*(*τ*)exp(−*ϕτ*); instead *g*(*τ*)exp(−*ϕτ*) needs to be renormalized to integrate to 1 because the generation-interval distribution is a probability distribution.

These two idealized interventions, in turn, allow us to understand how holding R or *r* fixed can lead to different conclusions about epidemic control. For example, if we assume epidemic strength R is known, then variation in the generation-interval distribution does not change the estimated effectiveness of a constant-strength intervention. In this same case, however, assuming longer generation intervals decreases the estimated epidemic growth rate (lower *r*), making an epidemic look easier to control with a constant-speed intervention. Conversely, when epidemic speed *r* is fixed, assuming longer generation intervals increases the estimated epidemic strength (R), making the epidemic look harder to control with a constant-strength intervention. Constant-strength and constant-speed interventions are idealized representations of real-life interventions, which can range from strength-like (e.g. vaccination and social distancing) to speed-like (e.g. contact tracing and isolation) depending on how their effectiveness varies across the generation interval (see [11] and Discussion).

Recent studies have suggested the possibility that new variants may have different generation-interval distributions. For example, Kissler *et al.* [33] suggested that the Alpha variant may have a longer duration of infection: 13.3 d (90% CI: 10.1–16.5), compared to 8.2 d (90% CI: 6.5–9.7) for the wild-type, thus suggesting that the mean generation interval of the Alpha variant is likely to be longer than that of the wild-type [22–24]. By contrast, some studies have suggested that the Delta variant may have a shorter generation interval due to faster within-host viral replication [34,35]. Modelling studies have also considered the possibility that the observed fast replacement of some variants may be driven, in part, by shorter generation intervals [2,6]. However, linking strength and speed is complicated given that the generation-interval distribution depends on many factors including behaviour: for example, self-isolation after symptom onset will lead to shorter generation intervals. The emergence of the Omicron variant, and its breakthrough infections in previously immune individuals, adds further uncertainty to individual-level transmission dynamics and therefore the generation interval of SARS-CoV-2 variants [36].

Here, we use the generation-interval-based framework to compare two measures of transmission advantage of new variants: the relative strength and relative speed. We assess how relative strength and speed depend on underlying epidemiological dynamics of previously dominant lineages and argue that assuming a constant relative strength (rather than a constant relative speed) is more appropriate for estimating relative transmissibility of new variants. We also show how neglecting differences in the generation-interval distributions of new variants can lead to biased estimates of their relative transmissibility and how such biases might be assessed in practice. Finally, we discuss how information on differences in generation interval distributions might influence priorities for controlling the spread of VoCs.

## 2. Renewal equation framework

We use the renewal equation framework to characterize the spread of two pathogen strains—in this case, the wild-type SARS-CoV-2 virus and a focal VoC. We focus on characterizing the incidence of infection, which is directly related to *r* and R. In practice, observed case reports are subject to reporting delays as well as changes in testing behaviours or capacity, which must be taken into account in order to correctly infer *r* and R [37,38].

Neglecting the (relatively slow) rate of new mutations and assuming homogeneous mixing, the current incidence of infection *i*_*x*_(*t*) caused by each strain *x*—either the wild-type (‘wt’) or the variant (‘var’)—can be expressed in terms of their previous incidence *i*_*x*_(*t* − *τ*) and the rate *K*_*x*_(*t*, *τ*) at which secondary infections are generated at time *t* by individuals infected *τ* time units ago:

2.1 $$i_{x}(t)=\int_{0}^{\infty }i_{x}(t-\tau )K_{x}(t,\tau )\;d\tau .$$

This framework provides a flexible way of modelling pathogen dynamics and generalizes a wide range of compartmental models, including the SEIR model [39–44].

Integrating the kernel at a fixed calendar time gives the instantaneous reproduction number:

2.2 $$R_{x}(t)=\int K_{x}(t,\tau )\;d\tau$$

which is defined as the expected number of secondary infections that would be caused by an individual infected at time *t* if conditions were to remain the same [45]. The instantaneous reproduction number is a particular kind of weighted average of infectiousness of previously infected individuals at time *t*—in particular, it is weighted by the total relative infectiousness at time *t*, rather than by the actual number of infected individuals present. The normalized kernel $g_{x}(t,\tau )=K_{x}(t,\tau )/R_{x}(t)$—which we refer to as the instantaneous generation-interval distribution—describes the relative contribution of previously infected individuals to current incidence *i*_*x*_(*t*) and provides information about the time scale of pathogen transmission. Like the instantaneous reproduction number, the instantaneous generation-interval distribution describes contributions to the epidemic under the counterfactual case where conditions remain constant at a particular set of values. Both the instantaneous reproduction number and the instantaneous generation-interval distribution depend on many factors, including intrinsic infectiousness of an infected individual, non-pharmaceutical interventions, awareness-driven behaviour and population-level susceptibility [45].

Constant-strength changes, which reduce transmission rate of infectious individuals independent of age of infection, do not change the instantaneous generation-interval distribution [45]. In this case, the instantaneous generation-interval distribution is also often referred to as the intrinsic generation-interval distribution [32,38,46,47]—for example, the standard SEIR model can be equivalently expressed as a renewal equation with time-invariant intrinsic generation-interval distribution *g*(*τ*) as shown in [44]. The instantaneous generation-interval distribution is different from the realized generation-interval distribution, which measures time between actual infection events [46]. Previous studies have noted, in many contexts, that the realized generation intervals can contract due to susceptible depletion—a special case of constant-strength changes [46,48,49]—even though the instantaneous generation-interval distribution remains unchanged in this scenario. While the instantaneous generation-interval distribution can change under speed-like changes (see [45] and §§6 and 7 for detailed discussions), assuming a time-invariant instantaneous generation-interval distribution is often appropriate in the context of SARS-CoV-2, given that control strategies against its spread have been primarily strength-like, including social distancing measures [50] and vaccination [51]. Indeed, many dynamical models of SARS-CoV-2 infections have solely relied on constant-strength changes, either implicitly or explicitly assuming a time-invariant intrinsic generation-interval distribution (e.g. [38,50,52]). Therefore, we neglect changes in the intrinsic generation-interval distribution over time for now and focus on the impact of constant-strength changes on the inference of dynamics of new SARS-CoV-2 variants. We revisit these ideas in §6 and compare the effects of constant-speed interventions with those of constant-strength interventions.

Over a short period of time, we can assume that epidemiological conditions remain roughly constant: $R_{x}(t)\approx R_{x}$ and *g*_*x*_(*t*, *τ*) ≈ *g*_*x*_(*τ*), in which case the incidence of infections changes exponentially. Here, we use the term ‘epidemiological conditions’ to broadly refer to all factors that affect transmission at the population level—mathematically, they are captured by the kernel *K*(*t*, *τ*). In the context of SARS-CoV-2 infections, we are essentially assuming that the changes in susceptible pool, behaviour, and contact rates are usually small over a short period of time—this assumption would not apply at the moment of a drastic policy change, but could be applied to the periods immediately before and after. Then, the incidence of each strain grows (or decays) exponentially at rate *r*_*x*_, satisfying the Euler–Lotka equation [15]:

2.3 $$\frac{1}{R_{x}}=\int_{0}^{\infty }\exp(-r_{x}\tau )g_{x}(\tau )\;d\tau .$$

We can approximate this *r*–R relationship by assuming that the generation-interval distribution is Gamma-distributed, and summarizing it using the mean generation interval ${\bar{G}}_{x}$ and the squared coefficient of variation *κ*_*x*_ (equal to the reciprocal of the Gamma shape parameter) [28]:

2.4 $$R_{x}\approx {\left(1+\kappa_{x}r_{x}{\bar{G}}_{x}\right)}^{1/\kappa_{x}}.$$

Various Gamma-generation-interval assumptions have been widely used in epidemic modelling, including for models of SARS-CoV-2 [53]. The Gamma-generation-interval assumption includes as a special case models that assume exponentially distributed generation intervals (when *κ* = 1), corresponding to the SIR model [10]. We note that when the infectious periods are Gamma distributed—another standard assumption in epidemic modelling—the resulting generation interval does not necessarily follow the Gamma distribution (see [24] for detailed discussion).

We use this framework to investigate how inferences about strength and speed of the variant depend on our assumptions about the underlying generation-interval distributions. Here, we focus on differences in mean generation intervals, assuming that both the variant and wild-type intervals are Gamma-distributed with squared coefficient of variation *κ*_wt_ = *κ*_var_ = *κ*. Changes in shape can be important [26,27], but we do not investigate them here. We do note that we expect a distribution with higher coefficient of variation to allow for more early transmission, and thus to have qualitatively similar effects to a distribution with a shorter mean during periods of growth, when early events are more important than later events [28].

## 3. Inferring relative strength from relative speed

When incidence is changing exponentially (*i*_*x*_(*t*) = *i*_*x*_(*t*_0_)exp(*r*_*x*_ *t*)), the proportion of the new variant *p*(*t*) follows a logistic growth curve [1,2]:

3.1 $$p(t)=\frac{i_{\mathrm{var}}(t_{0})\exp(r_{\mathrm{var}}t)}{i_{\mathrm{wt}}(t_{0})\exp(r_{\mathrm{wt}}t)+i_{\mathrm{var}}(t_{0})\exp(r_{\mathrm{var}}t)}\;$$

3.2 $$\;=\frac{1}{1+(i_{\mathrm{wt}}(t_{0})/i_{\mathrm{var}}(t_{0}))\exp(-\delta t)},$$

where the relative speed of the variant *δ* can be estimated as the slope of the log odds of *p* versus time. When more than two strains are co-circulating, the picture is more complicated [8]; we focus here on comparing two strains at a time.

We thus ask: what factors affect the relative strength $\rho =R_{\mathrm{var}}/R_{\mathrm{wt}}$ of a new variant, conditional on an observed relative speed *δ*? This inference depends on assumptions about the generation-interval distributions of both strains. Given the mean generation interval of the variant ${\bar{G}}_{\mathrm{var}}$ and the wild-type ${\bar{G}}_{\mathrm{wt}}$, the relative strength $\rho =R_{\mathrm{var}}/R_{\mathrm{wt}}$ under the Gamma assumption [28] is given by

3.3 $$\rho ={\left(\frac{1+\kappa (r_{\mathrm{wt}}+\delta ){\bar{G}}_{\mathrm{var}}}{1+\kappa r_{\mathrm{wt}}{\bar{G}}_{\mathrm{wt}}}\right)}^{1/\kappa }.$$

Therefore, the relative strength *ρ* depends not only on the relative speed *δ* and the generation-interval distributions but also on how fast the wild-type is spreading in the population (*r*_wt_)—some analyses have implicitly or explicitly neglected this factor by assuming either $r_{\mathrm{wt}}=0$ [1] or *κ* = 0 [2] (in the latter case, $\rho =\exp(\delta {\bar{G}}_{\mathrm{wt}})$ when ${\bar{G}}_{\mathrm{var}}={\bar{G}}_{\mathrm{wt}}$).

We start by taking the mean generation interval of the wild-type to be ${\bar{G}}_{\mathrm{wt}}=5\;d$ [54] and the squared coefficient of variation of generation intervals to be *κ* = 0.2 [54] for both the variant and the wild-type. As noted above, we assume throughout that the variant and the wild-type can be approximated with Gamma distributions with equal *κ*, and only consider differences in the mean. We evaluate the estimates of relative strength *ρ* across a wide range of *κ* from 0 (fixed-length generation intervals) to 1 (exponential distributions). To further explore how inference depends on underlying epidemiological conditions, we consider five scenarios, all with $\delta =r_{\mathrm{var}}-r_{\mathrm{wt}}=0.1\;d^{-1}$ (based on observations of the Alpha variant in the UK [2]), and with increasing underlying *r*: (1) $r_{\mathrm{wt}}<r_{\mathrm{var}}<0$, (2) $r_{\mathrm{wt}}<r_{\mathrm{var}}=0$, (3) $r_{\mathrm{wt}}<0<r_{\mathrm{var}}$, (4) $0=r_{\mathrm{wt}}<r_{\mathrm{var}}$ and (5) $0<r_{\mathrm{wt}}<r_{\mathrm{var}}$.

**Figure 1.**
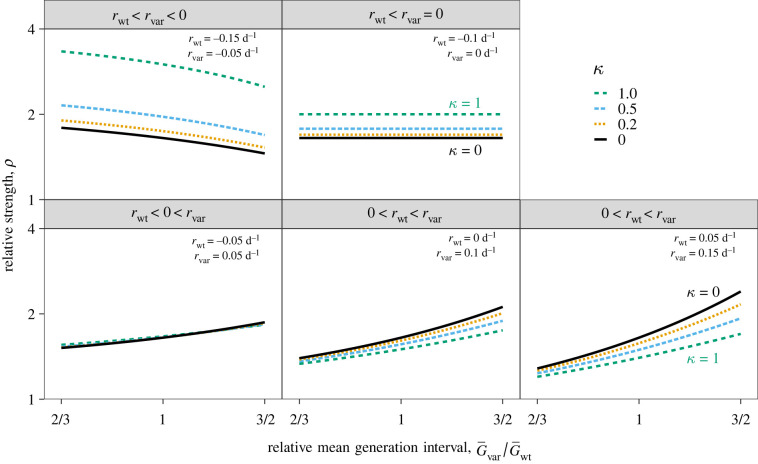
Relative strength of the new variant assuming a fixed speed advantage *δ* under five epidemiological conditions. The relative strength of the new variant *ρ* (shown on a log scale) conditional on the speed of the wild-type $r_{\mathrm{wt}}$; the ratio between the mean generation interval of the new variant ${\bar{G}}_{\mathrm{var}}$ and that of the wild-type ${\bar{G}}_{\mathrm{wt}}$; and the squared coefficient of variation in generation intervals *κ*. The relative strength of the new variant *ρ* is calculated using *δ* = 0.1 d^−1^, ${\bar{G}}_{\mathrm{wt}}=5\;d$ and *κ* = 1/5. Assumed values of $r_{\mathrm{wt}}$ and $r_{\mathrm{var}}$ are shown in the top right corner of each panel.

Unsurprisingly, we find that an increased speed of *δ* = 0.1 d^−1^ for the variant is consistent with higher strength (*ρ* > 1) than the wild-type across all five epidemiological scenarios considered (figure 1). However, the magnitude of relative strength *ρ* is sensitive to assumptions about generation intervals: for realistic values of *κ* (excluding 0 and 1), the inferred relative strength *ρ* ranges between 1.1 and 2.3 (across all five scenarios) when ${\bar{G}}_{\mathrm{var}}$ is allowed to vary between 2/3 and 3/2 of ${\bar{G}}_{\mathrm{wt}}$.

In general, longer mean generation intervals of the new variant translate to higher values of *ρ* (and vice versa; figure 1, bottom panels), except when $r_{\mathrm{var}}\leq 0$ (recall, we always assume $r_{\mathrm{wt}}<r_{\mathrm{var}}$; figure 1, top panels). When $r_{\mathrm{var}}=0$, we always have $R_{\mathrm{var}}=1$ and so *ρ* is independent of the generation-interval distribution of the new variant. When $r_{\mathrm{var}}<0$, we see that longer generation intervals decrease *ρ* because longer generation intervals actually lead to slower decay (higher *r*). Assuming a narrower distribution for both the variant and the wild-type strain (lower *κ*) has qualitatively similar effects to assuming longer generation intervals (leading to higher values of *ρ* when $r_{\mathrm{var}}>0$ and lower values of *ρ* when $r_{\mathrm{var}}<0$) because both reduce the amount of early transmission. When $r_{\mathrm{wt}}<0<r_{\mathrm{var}}$, inference of *ρ* is relatively insensitive to values of *κ*.

## 4. Inferring relative speed from relative strength

We do not generally expect the relative speed *δ* to remain fixed if other factors governing epidemic spread are changing. Instead, many biological mechanisms appear compatible with assuming a fixed value of relative strength *ρ* over changing conditions. For example, if the proportion of the population susceptible declines, or the average contact rate changes, while other factors remain unchanging, the relative strength *ρ* is expected to remain fixed [3,5,55,56].

We thus investigate how *δ* is expected to change with $R_{\mathrm{wt}}$ when *ρ* remains fixed, and how this expectation changes with the ratio of the generation intervals. Once again, we rely on the Gamma assumption [28] to find the relative speed *δ* given the mean generation interval of the variant ${\bar{G}}_{\mathrm{var}}$ and the wild-type ${\bar{G}}_{\mathrm{wt}}$:

4.1 $$\delta =\frac{{\left(\rho R_{\mathrm{wt}}\right)}^{\kappa }-1}{\kappa {\bar{G}}_{\mathrm{var}}}-\frac{R_{\mathrm{wt}}^{\kappa }-1}{\kappa {\bar{G}}_{\mathrm{wt}}}.$$

As our baseline scenario, we assume *ρ* = 1.61 (i.e. $R_{\mathrm{var}}=1.61R_{\mathrm{wt}}$), which is the value we obtain for *δ* = 0.1 d^−1^ [2], $r_{\mathrm{wt}}=0\;d^{-1}$, ${\bar{G}}_{\mathrm{wt}}={\bar{G}}_{\mathrm{var}}=5\;d$ and *κ* = 1/5 [54]. We evaluate *δ* across five scenarios as before: (1) $R_{\mathrm{wt}}<R_{\mathrm{var}}<1$, (2) $R_{\mathrm{wt}}<R_{\mathrm{var}}=1$, (3) $R_{\mathrm{wt}}<1<R_{\mathrm{var}}$, (4) $1=R_{\mathrm{wt}}<R_{\mathrm{var}}$ and (5) $1<R_{\mathrm{wt}}<R_{\mathrm{var}}$.

**Figure 2.**
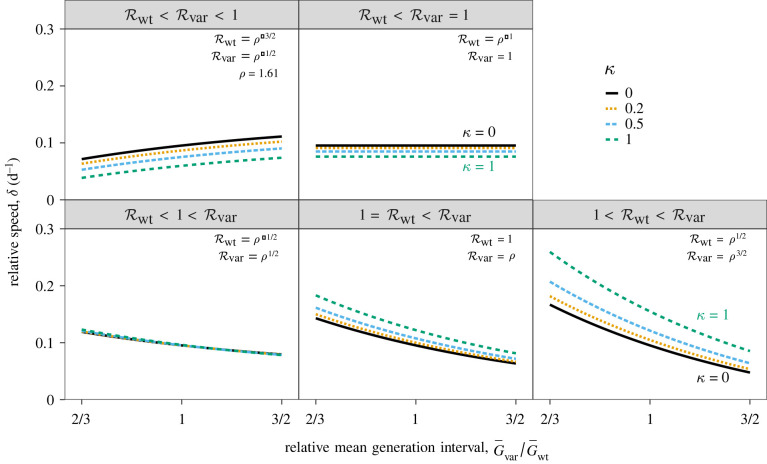
Relative speed of the new variant assuming a fixed strength advantage *ρ* under five epidemiological conditions. The relative speed of the new variant *δ* conditional on the strength of the wild-type $R_{\mathrm{wt}}$; ratio between the mean generation interval of the new variant ${\bar{G}}_{\mathrm{var}}$ and that of the wild-type ${\bar{G}}_{\mathrm{wt}}$; and squared coefficient of variation in generation intervals *κ*. Relative speed of the new variant *δ* is calculated using *ρ* = 1.61, ${\bar{G}}_{\mathrm{wt}}=5\;d$ and *κ* = 1/5. Assumed values of $R_{\mathrm{wt}}$ (and therefore $R_{\mathrm{var}}$) are shown in the top right corner of each panel.

In general, longer generation intervals lead to slower relative speed of the variant when the incidence of both strains is increasing (figure 2, bottom panels) because slower growth of the variant reduces the differences in absolute speed. When $R_{\mathrm{var}}=1$, the relative speed is insensitive to the generation-interval distribution of the variant because we always have $r_{\mathrm{var}}=0$ (figure 2, top-right panel). When $R_{\mathrm{var}}<1$, longer generation intervals of the variant lead to slower decay ($r_{\mathrm{var}}$ closer to 0), and therefore greater relative speed (figure 2, top-left panel). We see that assuming a narrower distribution for both the variant and the wild-type strain (lower *κ*) has qualitatively similar effects to assuming a longer mean (leading to lower values of *δ* when $R_{\mathrm{var}}>0$ and higher values of *δ* when $R_{\mathrm{var}}<0$).

Figure 2 also shows that when *ρ* is fixed, relative speed depends on underlying epidemiological conditions—specifically, the absolute strength of the two strains. For example, even when the generation-interval distributions are identical (${\bar{G}}_{\mathrm{var}}={\bar{G}}_{\mathrm{wt}}$, in this case), changing $R_{\mathrm{wt}}$ from 0.49 (figure 2, top-left panel) to 1.27 (figure 2, bottom-right panel)—and thus $R_{\mathrm{var}}$ from 0.79 to 2.04—changes the relative speed *δ* from 0.08 to 0.11 when *κ* = 0.2 (and from 0.06 to 0.14 when *κ* = 0.5). Differences in the generation-interval distributions exaggerate these changes. Therefore, characterizing changes in the proportion of variants by assuming a fixed relative speed (e.g. by fitting a standard logistic growth curve [1] and using the resulting value of *δ*) should be done with care.

## 5. Inferring relative strength from incidence data

Instead of inferring relative strength from relative speed, one can separately estimate time-varying reproduction numbers R(t) of the variant and the wild-type from observed incidence and assumptions about the generation intervals, and then calculate the ratio—such methods have been used in previous analyses of the Alpha [6], Delta [57] and Omicron variants [58]. Broadly, there are two types of time-varying reproduction numbers: case reproduction number and instantaneous reproduction number. The case reproduction number is defined as the average number of secondary infections caused by an individual infected at time *t* and therefore depends on transmission after time *t* [59]. The instantaneous reproduction number is defined as the average number of secondary infections that would be caused by an individual infected at time *t* if conditions were to remain the same [45]; therefore, the instantaneous reproduction number only depends on transmission at time *t* and is most appropriate for real-time evaluation of changes in transmission [38]. The estimation of the instantaneous reproduction number was popularized by Cori *et al*. [60] and has been widely adopted in epidemiological analyses of SARS-CoV-2 [50,52,61–63].

In practice, estimating the instantaneous reproduction number is complicated because it requires estimating incidence of infection. Observed case counts are sensitive to reporting delays [37] and changes in case definitions [64], which in turn can affect estimates of the instantaneous reproduction number [38]. Here, we choose to focus on the underlying dynamical mechanisms that may affect inference and thus assume that the incidence of infection is known exactly. Assuming that the instantaneous generation-interval distribution remains constant, the instantaneous reproduction numbers of the new variant and of the wild-type can be estimated from their corresponding incidence curves [60]:

5.1 $$R_{x}(t)=\frac{i_{x}(t)}{\int_{0}^{\infty }i_{x}(t-\tau )g_{x}(\tau )\;d\tau }.$$

Under constant-strength intervention measures that reduce transmission rates of both strains by a constant amount, we expect ratios between reproduction numbers to remain constant and correspond to the true relative strength: $R_{\mathrm{var}}(t)/R_{\mathrm{wt}}(t)=\rho$ [3,5,55,56]. However, if the assumed generation-interval distribution $\hat{g}(\tau )$ differs from the true distribution, then the ratio between the estimated reproduction numbers $\hat{\rho }(t)={\hat{R}}_{\mathrm{var}}(t)/{\hat{R}}_{\mathrm{wt}}(t)$ may change, even if the true ratio does not.

**Figure 3.**
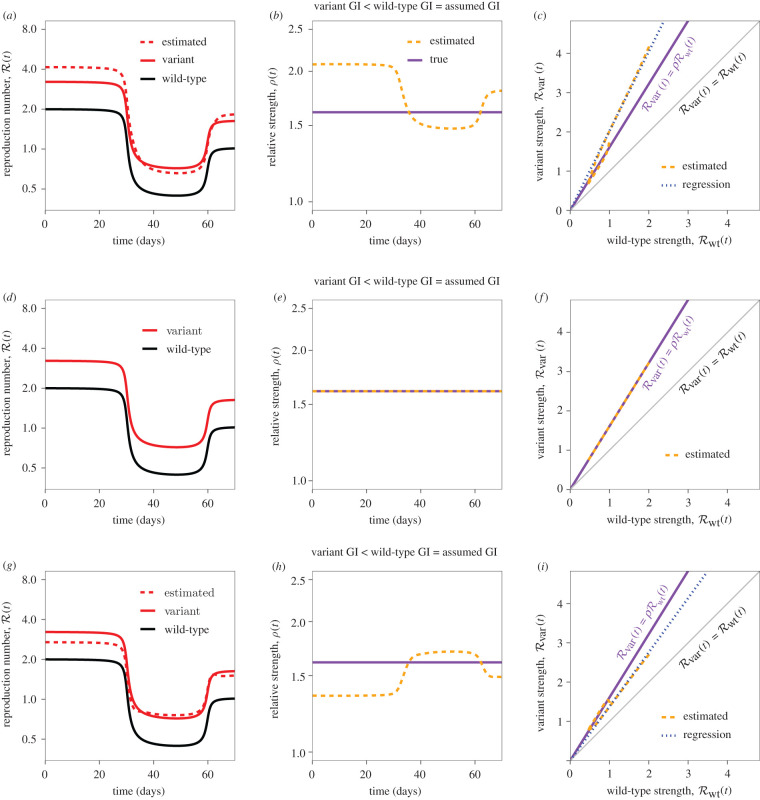
Estimates of relative strength over time under different scenarios. (*a*,*d*,*g*) True (solid lines) and estimated (dashed lines) reproduction numbers of the new variant and the wild-type over time. (*b*,*e*,*h*) True (purple, solid) and estimated (orange, dashed) ratios between reproduction numbers of the new variant and the wild-type over time. (*c*,*f*,*i*) Phase planes (time is implicit) showing true (purple, solid) and estimated (orange, dashed) relationships between estimated reproduction numbers. Blue dotted lines represent the regression lines of the estimated variant reproduction numbers against the estimated wild-type reproduction numbers. Grey lines represent the one-to-one line. For all simulations, the assumed mean generation interval is equal to the mean generation interval of the wild-type (5 d), and the squared coefficient of variation in generation intervals is equal to *κ* = 1/5. The mean generation interval of the variant is equal to 4 d (*a*–*c*), 5 d (*d*–*f*) and 6 d (*g*–*i*).

Here, we investigate how misspecification of the generation-interval distribution of the variant affects our inference of relative strength from incidence data under the assumption that the true generation-interval distribution of the wild-type is known. We use a two-strain renewal equation that assumes perfect cross-immunity to simulate three different scenarios (see electronic supplementary material, Text): (1) the variant has a shorter mean generation interval (figure 3*a*–*c*); (2) the wild-type and the variant have the same (known) generation-interval distributions (figure 3*d*–*f*); and (3) the variant has a longer mean generation interval (figure 3*g*–*i*). Then, we compare the estimated ratio $\hat{\rho }(t)={\hat{R}}_{\mathrm{var}}(t)/{\hat{R}}_{\mathrm{wt}}(t)$ with the true ratio $\rho =R_{\mathrm{var}}(t)/R_{\mathrm{wt}}(t)$. In order to simulate introduction and lifting of non-pharmaceutical interventions, we let $R_{\mathrm{wt}}(t)$ decrease from 2 to 0.4 around day 30 and increase back up to 1 around day 60 and assume $R_{\mathrm{var}}(t)=\rho R_{\mathrm{wt}}(t)$. Previous studies have modelled the impact of non-pharmaceutical interventions as a step function [50], but we use a smooth function to model $R_{\mathrm{wt}}(t)$ (figure 3; see electronic supplementary material, Text) given the possibility that behavioural changes may affect transmission before and after interventions take place. We reach similar conclusions if we use a step function instead (electronic supplementary material, figure S1).

When the wild-type and the variant have the same (known) generation-interval distributions, the assumed generation-interval distribution matches the true distribution (figure 3*d*–*f*). In this case, the estimated reproduction numbers match the true values (figure 3*d*); thus, their estimated ratio $\hat{\rho }(t)$ remains constant and ${\hat{R}}_{\mathrm{var}}(t)=R_{\mathrm{var}}(t)$ (figure 3*e*). However, when the true generation intervals of the variant have a shorter mean than the assumed distribution, we over-estimate $R_{\mathrm{var}}(t)$ during the growth phase and under-estimate $R_{\mathrm{var}}(t)$ during the decay phase (figure 3*a*); therefore, the estimated ratio $\hat{\rho }(t)$ changes over time (figure 3*b*). Conversely, when the true generation intervals of the variant have a longer mean than the assumed distribution, we find similar biases in opposite directions (figure 3*g*,*h*).

In practice, estimates of instantaneous reproduction numbers R(t) (and therefore, their ratios) can be noisy due to limited data availability or model assumptions; instead, we might want to estimate a single value of relative strength *ρ*. For example, we can estimate *ρ* by plotting the estimated strength of the variant ${\hat{R}}_{\mathrm{var}}(t)$ against the estimated strength of the wild-type ${\hat{R}}_{\mathrm{wt}}(t)$—as presented in fig. 2 of [6]—and performing a linear regression (figure 3*c*,*f*,*i*). Here, we estimate the slope while fixing the intercept to zero: the regression line should go through the origin in theory because $R_{\mathrm{var}}=0$ when $R_{\mathrm{wt}}=0$. If *ρ* is constant, and generation-interval distributions are correctly specified, we obtain a straight line with a slope of *ρ* and intercept at zero (figure 3*f*). However, when the assumed mean generation interval is longer than that of the variant (figure 3*c*), we over-estimate the slope (and conversely, figure 3*i*).

## 6. Implications for intervention strategies

While relative speed *δ* and strength *ρ* are useful for characterizing the spread of the variant in an epidemiological context with a previously dominant wild-type, the *absolute* speed $r_{\mathrm{var}}$ and strength $R_{\mathrm{var}}$ of the variant determine the spread and conditions for control of the variant over the long term. In particular, at any given point in the epidemic, we can measure the speed of the variant $r_{\mathrm{var}}$ (or infer $r_{\mathrm{var}}$ from $r_{\mathrm{wt}}$ and *δ*) and ask how much more intervention is required to control the spread of the variant (and thus also the wild-type, which is assumed to be easier to control). As a baseline scenario, we assume $r_{\mathrm{wt}}=0$ and *δ* = 0.1 d^−1^ (and therefore $r_{\mathrm{var}}=0.1\;d^{-1}$), in which case additional intervention is required to reduce $r_{\mathrm{var}}$ below 0 (or, equivalently, $R_{\mathrm{var}}$ below 1).

**Figure 4.**
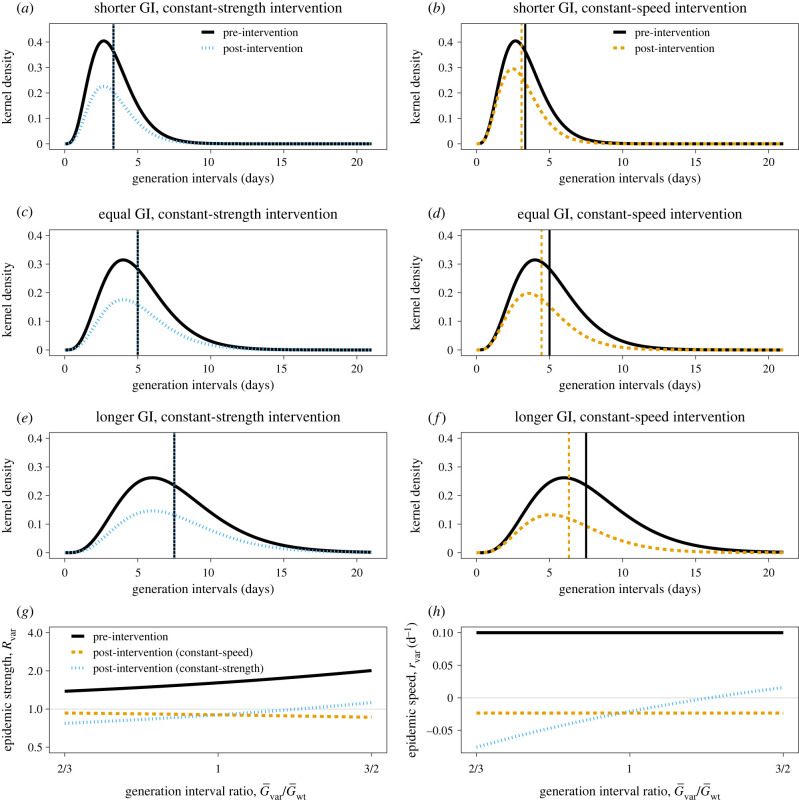
Effects of constant-strength and constant-speed interventions on the spread of a new variant with known speed *r*_var_. (*a*–*f*) Pre-intervention (black) and post-intervention (coloured) kernel of the new variant under constant-strength (*a*,*c*,*e*) and constant-speed (*b*,*d*,*f*) interventions when the variant has shorter (*a*,*b*), equal (*c*,*d*), or longer (*e*,*f*) mean generation interval (GI) than the wild-type. Vertical lines represent the mean generation interval. (*g*) Pre-intervention $R_{\mathrm{var}}$ (black) and post-intervention epidemic strength $R_{\mathrm{post}}$ (coloured) conditional on the mean generation interval of the variant. (*h*) Pre-intervention $r_{\mathrm{var}}$ (black) and post-intervention epidemic speed $R_{\mathrm{post}}$ (coloured) conditional on the mean generation interval of the variant. Epidemic strength and speed are calculated assuming $r_{\mathrm{wt}}=0\;d^{-1}$, *δ* = 0.1 d^−1^, $r_{\mathrm{var}}=0.1\;d^{-1}$, ${\bar{G}}_{\mathrm{wt}}=5\;d$ and *κ* = 1/5 for pre-intervention conditions. The constant-strength intervention assumes that the ratio $R_{\mathrm{var}}/R_{\mathrm{post}}=\theta$ remains constant. The constant-speed intervention assumes that the difference $r_{\mathrm{var}}-R_{\mathrm{post}}=\phi$ remains constant. Intervention strength and speed are chosen so that post-intervention strength of the new variant is 0.9 when its mean generation interval is 5 d.

We consider two types of intervention: an intervention of constant strength (figure 4*a*,*c*,*e*), which reduces transmission by a constant factor *θ* regardless of age of infection (*K*_post_(*τ*) = *K*_pre_(*τ*)/*θ*); and an intervention of constant speed (figure 4*b*,*d*,*f*), which reduces transmission after infection by a constant rate *ϕ* (*K*_post_(*τ*) = *K*_pre_(*τ*)exp(−*ϕτ*)); both of these interventions are constant across generation intervals, but not necessarily across calendar time. In this case, we can control the spread of the variant when $\theta >R_{\mathrm{var}}$ or $\phi >r_{\mathrm{var}}$, respectively [11]. We consider constant-strength and -speed interventions calibrated to reduce $R_{\mathrm{var}}$ to 0.9 under the assumption that the variant generation intervals match those of the wild-type (figure 4*c*,*d*). While both interventions are equally effective on the strength scale (that is, $R_{\mathrm{post}}=\int K_{\mathrm{post}}(\tau )\;d\tau =0.9$), they have different dynamical implications. The constant-strength intervention affects transmission equally throughout the course of infection, whereas the constant-speed intervention has greater impact on transmission that occurs later in infection; as a result, the constant-speed intervention reduces the post-intervention mean generation interval (figure 4*b*) and leads to (slightly) faster exponential decay (therefore, lower $R_{\mathrm{post}}$).

However, if the variant has longer generation intervals than the wild-type (figure 4*e*,*f*), then the strength of the variant will be higher because we are holding the observed speed constant (figure 4*g*). In this case, the same constant-strength intervention can fail to control the epidemic (i.e.$R_{\mathrm{post}}>1$; figure 4*h*) because this intervention reduces the transmission by a constant amount regardless of age of infection (figure 4*e*). On the other hand, the same constant-speed intervention will prevent a larger proportion of transmission, leading to lower $R_{\mathrm{post}}$ (figure 4*g*), because it is more effective against late-stage transmission (figure 4*f*). The constant-speed intervention also reduces the mean generation interval by a larger factor (figure 4*f*). Conversely, if the variant has shorter generation intervals than the wild-type (figure 4*a*,*b*), the given constant-strength intervention will be relatively more effective (figure 4*a*) because the given constant-speed intervention prevents less transmission (figure 4*b*).

The speed-based paradigm gives the same results regarding control but provides additional insight (figure 4*f*). The observed speed of the variant $r_{\mathrm{var}}$ at a given moment is independent of our estimates of its mean generation interval. Likewise, the post-intervention speed of the variant under the constant-speed intervention is also independent of the mean generation interval. Therefore, if speed of intervention is faster than the observed speed of spread (i.e. if $\phi >r_{\mathrm{var}}$), we can control the epidemic (i.e. $R_{\mathrm{post}}<0$) regardless of the underlying generation-interval distribution (see [11] for mathematical details).

**Figure 5.**
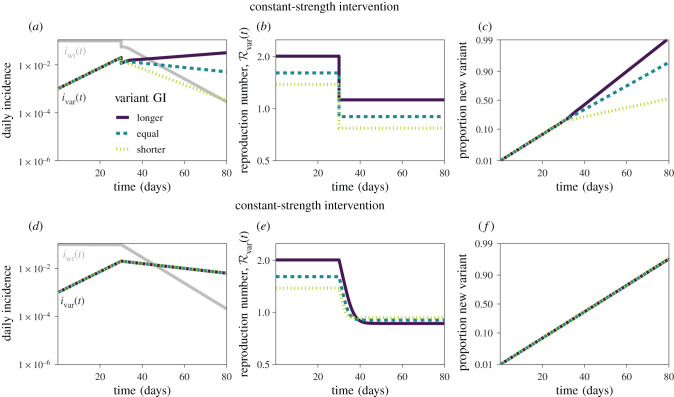
Simulations of constant-strength and constant-speed interventions. Constant-strength (*a*–*c*) and constant-speed (*d*–*f*) interventions are simulated using the renewal equation (see electronic supplementary material for details of simulations). (*a*,*d*) Daily incidence of infections caused by the wild-type ($i_{\mathrm{wt}}(t)$, grey) and the variant (*i*_var_(*t*), coloured). (*b*,*e*) Instantaneous reproduction number of the variant $R_{\mathrm{var}}(t)$ calculated using equation (2.2). (*c*,*f*) Proportion of incidence of infections caused by the variant on a logit scale. Epidemic trajectories are calculated assuming $r_{\mathrm{wt}}=0\;d^{-1}$, *δ* = 0.1 d^−1^, $r_{\mathrm{var}}=0.1\;d^{-1}$, ${\bar{G}}_{\mathrm{wt}}=5\;d$ and *κ* = 1/5 for pre-intervention conditions, and then introducing constant-strength and constant-speed interventions on day 30. The pre-intervention ratio between the mean generation interval ${\bar{G}}_{\mathrm{var}}/{\bar{G}}_{\mathrm{wt}}$ is assumed to equal 1.5 (longer), 1 (equal) and 1/1.5 (shorter) for three different scenarios. The constant-strength and constant-speed interventions are identically modelled as illustrated in figure 4.

Finally, we synthesize our findings and illustrate the differences between constant-strength and constant-speed interventions using epidemic simulations (figure 5). As before, we consider a scenario in which a new variant is emerging with known relative speed ($\delta =r_{\mathrm{var}}>0.1\;d^{-1}\Rightarrow R_{\mathrm{wt}}>1$) while the incidence of infections caused by the wild-type is constant ($r_{\mathrm{wt}}=0\;d^{-1}\Rightarrow R_{\mathrm{wt}}=1$) before interventions are introduced. The constant-strength intervention then reduces both $R_{\mathrm{wt}}$ and $R_{\mathrm{var}}$ by a factor *θ* beginning on day 30, whereas the constant-speed intervention isolates individuals infected with both the wild-type and the variant at a constant hazard *ϕ* after day 30. If the variant has a longer mean generation interval, this particular constant-strength intervention fails to suppress the spread of the variant (figure 5*a*) because the longer generations imply a higher initial $R_{\mathrm{wt}}$, and thus a stronger intervention is required to reduce $R_{\mathrm{wt}}$ below 1 (figure 5*b*). Conversely, if the variant has a shorter mean generation interval, its initial $R_{\mathrm{wt}}$ will be lower, and therefore the same constant-strength intervention will be more effective, leading to lower post-intervention $R_{\mathrm{wt}}$ (figure 5*b*). The relative strength remains constant under constant-strength intervention (figure 5*b*), but the relative speed changes (figure 5*c*) as discussed earlier in §4—in this case, the longer mean generation interval variant increases the relative speed when the intervention is introduced.

In contrast, epidemic trajectories under the constant-speed intervention behave identically regardless of the mean generation interval of the variant (figure 5*d*). As shown in figure 4*e*, a constant-speed intervention leads to greater reduction in $R_{\mathrm{var}}(t)$ when the mean generation interval of the variant is longer (figure 5*e*)—this implies a change in relative strength *ρ* because the resulting post-intervention strength under a constant-speed intervention is sensitive to the generation-interval distribution as shown in figure 4. In this case, the relative speed of the variant remains unaffected by the constant-speed intervention because the epidemic speed of the wild-type $r_{\mathrm{wt}}$ and the variant $r_{\mathrm{var}}$ are both reduced by equal amounts *ϕ*, resulting in the post-intervention epidemic speed of $r_{\mathrm{wt}}-\phi$ and $r_{\mathrm{var}}-\phi$, respectively.

## 7. Discussion

We explored how the generation-interval distribution shapes the link between relative strength and speed. While the role of generation-interval distributions in linking epidemic strength and speed is well established, this framework also provides insight into an under-appreciated phenomenon: the relative strength and speed of new variants also depend on both current epidemic conditions and the nature of proposed control measures (e.g. strength- or speed-like) to be introduced. For example, under a constant-strength intervention that reduces the transmission rate of the wild-type and a variant by the same amount (and therefore keeps the relative strength constant), the relative speed of the variant depends on the epidemic growth rate of the wild-type strain. Our results challenge the assumption commonly made when characterizing changes in the proportion of new variants that the relative speed of new variants remains constant over time.

Differences in the generation-interval distributions further exaggerate this effect. Therefore, neglecting potential differences in the mean generation intervals between the variant and the wild-type can bias estimates of the variant’s transmission advantage. These biases may be assessed by considering whether estimates of relative strength appear to vary systematically with the direction of changes in the incidence of infections caused by the variant. Differences in generation intervals can also lead to different conclusions about the effectiveness of interventions. If the variant has longer generation intervals than the wild-type, speed-like interventions will be relatively more effective than naive estimates would suggest. Conversely, strength-like intervention will be relatively more effective if the variant has shorter generation intervals.

This perspective sheds light on earlier estimates of relative strength of the Alpha variant. Mathematical analyses typically reported greater than a 1.4-fold increase in reproduction number for the Alpha variant (*ρ* > 1.4), whereas an independent analysis of secondary attack rates from contact tracing data suggested a somewhat smaller 1.25- to 1.4-fold increase [65]. As shown in [2,6], propagating uncertainty in generation interval estimates (and, in particular, assuming shorter generation intervals) partially explains these differences: for example, if we consider a generation-interval distribution estimate with a short mean and wide variability from Tianjin, China (${\bar{G}}_{\mathrm{wt}}={\bar{G}}_{\mathrm{var}}=2.57\;d$ and *κ* = 1 [66]), we obtain *ρ* = 1.26 (from *δ* = 0.1 d^−1^ and $r_{\mathrm{wt}}=0\;d^{-1}$). Although these estimates are more consistent with the attack rate analysis [65], they are necessarily more accurate. While individual-level data based on contact tracing can provide a more reliable source of information about the transmissibility and time scale of transmission in some cases, they can also be biased towards particular types of contacts—for example, household contacts are more likely to be identified—which could also affect the estimate of *ρ*. Instead, this calculation simply highlights the importance of carefully considering generation-interval distributions in assessing the relative strength and speed of SARS-CoV-2 variants. We relied on parameter estimates for the Alpha variant throughout our analysis, but our qualitative conclusions can be applied to studying other SARS-CoV-2 variants, including the Delta and Omicron variants.

Other studies have also used generation-interval-based arguments to explore changes in estimates of relative strength. For example, Volz *et al.* estimated that the relative strength of the Alpha variant declined in England between December 2020 and January 2021 [6]. They hypothesized that a shorter generation interval of the Alpha variant could explain this phenomenon by reducing the Alpha variant’s relative speed (and therefore its relative strength) under intervention measures. However, our analysis suggests that a shorter generation interval of a new variant cannot explain the decline in relative strength. Under a constant-strength intervention, the relative speed decreases (as predicted by [6]) but the relative strength remains constant (figure 5*b*). Under a constant-speed intervention, the relative speed remains constant (figure 5*f*), but the relative strength increases because the intervention will have less effect on the variant with a shorter generation interval (figure 5*e*).

We have focused on the differences in the mean generation interval, but differences in the amount of variability, characterized by the squared coefficient of variation *κ*, could also have important, but different implications [67]. For example, if two variants have identical reproduction numbers, the variant with a wider generation-interval distribution will always out-compete the other variant (except when both variants have R=1) because a variant with a wider generation-interval distribution can take advantage of more early transmission during the growth phase and more late transmission during the decay phase.

We further used simulations to show how misspecification of generation-interval distributions can bias the inference of relative strength from incidence data. In doing so, we assumed that the intervention would reduce transmission caused by the variant and the wild-type by equal amounts, thereby preserving the relative strength over time; however, this assumption only holds under strength-like interventions, which are insensitive to time since infection, but not under speed-like interventions. As we demonstrated, if the variant and the wild-type have different generation intervals, speed-like interventions such as contact tracing can affect them differently, causing their relative strength to change over time (but not necessarily their relative speed).

We mostly considered idealized interventions of constant strength or constant speed. Real interventions are likely more complex. Interventions that reduce contact rates, such as social distancing measures, are generally strength-like, but they can also elicit awareness-based behavioural responses that are speed-like—for example, infected individuals may self-isolate faster after symptom onset. On the other hand, the hazard of isolation-based interventions (and thus, speed-like), such as contact tracing, can vary over the course of infection depending on delays in tracing infected individuals [54,68,69]. Their effectiveness can further depend on the ‘coverage’ of isolation, which is strength-like—for example, there may be a group of individuals that cannot be isolated by certain interventions due to asymptomaticity or the lack of participation in the contact tracing programme. Further, some interventions, such as vaccination, can also have disproportionate different effects on different variants—another complication not considered in our analysis.

Analyses of SARS-CoV-2 dynamics have primarily relied on the constant-strength framework [38,52,70], even when modelling speed-like interventions such as self-isolation [50,71]. While the constant-strength framework provides a convenient tool for modelling and understanding epidemic dynamics, we encourage researchers to consider both strength- and speed-based perspectives, as they can lead to different conclusions.

This study has practical implications for analysing the epidemiological dynamics of new variants. First, models that assume time-invariant relative speed, such as the standard logistic growth model, should be used with care—it is important to remember that the relative speed is expected to change with epidemiological conditions. Early in the spread of an emerging variant, it is likely more convenient to fix the relative speed, given that speed can be directly observed. However, epidemiological conditions *will* change over time in response to spreading new variants—in the context of SARS-CoV-2 infections, most of these responses (e.g. vaccination and social distancing) have been strength-like, causing relative speed to change and invalidating this assumption. As more information about the transmission and immunity profiles of the new variant becomes available, we advise instead fixing the relative strength and inferring the speed, as this assumption better matches biological mechanisms for the variants’ higher strength (e.g. higher rates of transmission and immune evasion). However, as interventions can often have both strength- and speed-like aspects, both the relative strength and speed can vary at the same time. Second, the absolute strength and speed should not be neglected in favour of relative values. While the relative strength and speed are useful for describing the spread of new variants, the absolute values determine their spread and control. Finally, uncertainty in generation intervals should be carefully considered.

While detailed contact tracing data can help narrow down uncertainties in the generation-interval distributions, there are additional complexities to comparing generation-interval estimates of different variants. The generation intervals can be measured in two different ways: backward and forward [46]. The backward distribution starts from the cohort of infectees who were infected at the same time and looks at when their infectors were infected. The backward distribution is subject to dynamical biases because we are more likely to observe recent infections (and therefore shorter generation intervals) when the epidemic is growing. On the other hand, the forward distribution starts from the cohort of infectors who were infected at the same time and looks at when they transmitted infections. While the forward distribution is more stable, changes in epidemic dynamics due to intervention measures and susceptible depletions can affect the shape of either distribution [20,49]. For example, comparing generation-interval estimates from different intervention periods will necessarily bias the true differences in the generation-interval distributions of different variants.

Even though SARS-CoV-2 has been spreading for more than a year, there is still considerable uncertainty about its generation intervals. Several studies have tried to estimate the generation-interval distribution, with means ranging between 3 and 6 d and squared coefficients of variation ranging between 0.1 and 1 [54,62,66,72]. However, these estimates are derived from serial intervals (i.e. time between symptom onset of the infector and the infectee [16]), which are subject to dynamical biases [21] and fail to account for asymptomatic transmission [32]. A recent study has further suggested that the generation intervals of the original SARS-CoV-2 strain may be considerably longer than previously thought [73], adding further uncertainty. A few studies have tried to compare generation- and serial-interval distributions of new SARS-CoV-2 variants with those of the original wild-type strain [74,75]; these comparisons are inherently difficult due to temporal changes in generation- and serial-interval distributions caused by dynamical and intervention effects [20,21]. Future studies should prioritize detailed assessment of the generation intervals of SARS-CoV-2 and widespread variants, as well as consider how uncertainty in generational intervals might bias conclusions [20,38,53]. Combining sequencing or viral load trajectory data can also help narrow estimates of generation- and serial-interval distributions as well as time-varying reproduction numbers even when contact tracing data are limited [76,77].

The spread of new SARS-CoV-2 variants and the replacement of previously dominant lineages represent ongoing challenges for controlling the SARS-CoV-2 pandemic [36,78–80]. By explicitly considering epidemiological context and generation-interval differences together, we have shown that improving estimates of the relative duration of infectiousness at the individual scale may help guide more effective interventions. Specifically, speed-like interventions, such as contact tracing, will be relatively more effective if variants have longer generation intervals. Most intervention strategies throughout the current pandemic have focused on strength-like interventions [50], such as lock-downs, partly because pre-symptomatic transmission of SARS-CoV-2 has limited the effectiveness of contact tracing efforts [81]. However, given the possibility that new variants can have different infection characteristics, future studies should consider whether their transmission dynamics also differ (e.g. the amount of pre-symptomatic transmission) and evaluate intervention strategies accordingly.

## Data Availability

All data and code are stored in a publicly available GitHub repository (https://github.com/parksw3/newvariant) [82]. Electronic supplementary material is available online [83].
